# Nanoconfined Fluids: Uniqueness of Water Compared to Other Liquids

**DOI:** 10.1021/acsnano.1c07381

**Published:** 2021-11-22

**Authors:** Fabio Leoni, Carles Calero, Giancarlo Franzese

**Affiliations:** † Department of Physics, Sapienza University of Rome, Piazzale Aldo Moro 5, 00185 Rome, Italy; ‡ Secció de Física Estadística i Interdisciplinària-Departament de Física de la Matèria Condensada, Institut de Nanociència i Nanotecnologia (IN2UB), Universitat de Barcelona, Carrer Martí i Franquès 1, 08028 Barcelona, Spain

**Keywords:** confinement effects, graphene, simple and anomalous liquids, water hydrogen-bond network, diffusion, hydration pressure, free energy

## Abstract

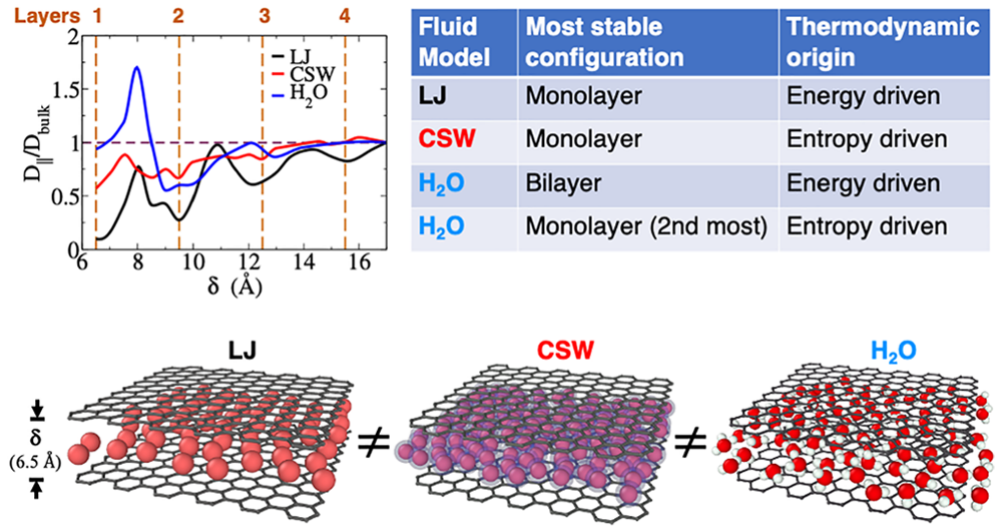

Nanoconfinement can drastically change the behavior of liquids, puzzling us with counterintuitive properties. It is relevant in applications, including decontamination and crystallization control. However, it still lacks a systematic analysis for fluids with different bulk properties. Here we address this gap. We compare, by molecular dynamics simulations, three different liquids in a graphene slit pore: (1) A simple fluid, such as argon, described by a Lennard-Jones potential; (2) an anomalous fluid, such as a liquid metal, modeled with an isotropic core-softened potential; and (3) water, the prototypical anomalous liquid, with directional HBs. We study how the slit-pore width affects the structure, thermodynamics, and dynamics of the fluids. All the fluids show similar oscillating properties by changing the pore size. However, their free-energy minima are quite different in nature: (i) are energy-driven for the simple liquid; (ii) are entropy-driven for the isotropic core-softened potential; and (iii) have a changing nature for water. Indeed, for water, the monolayer minimum is entropy driven, at variance with the simple liquid, while the bilayer minimum is energy driven, at variance with the other anomalous liquid. Also, water has a large increase in diffusion for subnm slit pores, becoming faster than bulk. Instead, the other two fluids have diffusion oscillations much smaller than water, slowing down for decreasing slit-pore width. Our results, clarifying that water confined at the subnm scale behaves differently from other (simple or anomalous) fluids under similar confinement, are possibly relevant in nanopores applications, for example, in water purification from contaminants.

Fluids under nanoconfinement are challenging to understand because they can show properties that are quite different compared to their bulk counterpart.^1−12^ For example, they form layers parallel to the confining surfaces,^13^ and, when the confinement width is ultrathin, the layers can solidify in peculiar structures.^14^ In the case of nanoconfined water, freezing can happen both above^15−17^ or below^18,19^ the bulk melting temperature depending on the confining system. Simulations of a monatomic water model nanoconfined to form only two layers show even dynamical oscillations between the liquid phase and ice.^20^ Nanoconfined fluids are relevant for their implications in life science and nanotechnology^5,21−38^ and for applications such as purification of fluids forced through microporous carbon materials^39−41^ nanolubrication^42^ or isotope separation in nuclear power technology.^43^ The fabrication of nanoscale membranes^44^ allows to investigate transport properties at the molecular level, revealing fast permeation of water through carbon nanotubes^45−47^ and through graphene oxide membranes,^48^ which can be used for filtration of complex mixtures and water disinfection and desalination.

Confined fluids have been studied extensively by numerical simulations in various geometries, including surfaces or slit, tubular, and cubic pores, with flat or rough walls or with different wall permeabilities, finding relationships between pore size and selectivity.^49^ In particular, computer simulations show that nanoconfinement may influence the dynamical properties of fluids. For example, water diffusivity is enhanced when the confining slit pore formed by hexagonal boron nitride sheets allows the formation of one or two layers.^50^ Also, liquid films of nonpolar molecules, confined between two solid walls, undergo an abrupt change in the diffusion constant and support shear,^51,52^ or freeze to a solid as the structured wall,^53^ when the confinement reduces to a few molecular layers. Experiments confirm the liquid-to-solid transition for simple organic solvents (cyclohexane, octamethylcyclotetrasiloxane, and toluene) under confinement when decreasing from seven to six molecular layers.^54,55^

Molecular dynamics (MD) simulations of a Lennard-Jones (LJ) liquid in slit pores, with widths from 2 to 12 molecular diameters and structureless walls, show a weak increase of the local parallel diffusion for the particles initially within the first layer near the wall.^56^ By varying pore width at constant chemical potential, both parallel diffusion coefficient and solvation force oscillate and saturate to the bulk value for widths >10 molecular diameters.^57,58^ Moreover, for both a LJ liquid^59^ and a LJ binary equimolar mixture,^60^ the self-diffusion coefficient reduces when the confining scale decreases or the interaction of the fluid with the walls increases. However, these results are at variance with those for simple gases confined in carbon nanotubes, where the diffusion coefficient is larger for smaller nanotube diameters.^61−64^

Also, for anomalous liquids^65^ and water in carbon nanotubes, the diffusion coefficient changes in a nonmonotonic way and the flow can be enhanced^66−68^ with decreasing nanotube diameters,^69^ especially for diameters below 1 nm.^22,70−72^ On the other hand, previous simulations of water confined in nanotube of different diameters show that the diffusion along the axes decreases for smaller diameters.^73^

Contradictory results have been found also for the shear viscosity of water confined in a graphene nonotube. It monotonically increases for increasing channel diameter^74,75^ or oscillates and decreases for increasing slit pore width, depending on the specific water model.^76^ Finally, density functional theory calculations^77^ and Monte Carlo simulations^78^ suggest that the SPC/E model of water and simple liquids like LJ behave similarly when confined by a single surface.

It is, therefore, worth asking how these varieties of different results depend on the details of the fluid interactions or the confining geometry. For example, Striolo finds a relevant difference between the diffusion of simple fluids and water in molecular sieves.^79^ While the first is dominated by concerted events in which multiple molecules move simultaneously due to the spatial mismatches between pore–fluid and fluid–fluid attractive interactions, the ballistic diffusion of water clusters is a consequence of long-lasting hydrogen bonds (HBs).^79^

Here, we deepen this question and ask which property of water nanoconfined in a graphene-like slit pore is distinctive and which is shared with other anomalous liquids or even normal liquids. To this end, we perform MD simulations of the LJ fluid and the continuous shouldered well (CSW) anomalous fluid^80−84^ under slit-pore confinement. The CSW fluid is a coarse-grained model for fluids, including liquid metals or complex liquids,^85^ with water-like properties associated with the presence of two length scales,^81,82^ such as the hydrophobic effect.^86^ It is used, also, to model hydroxyl groups interactions in methanol^87−89^ and water–hydroxyl groups interactions in water/methanol mixtures.^90^ A potential similar to the CSW has been used to study the effect of macromolecular crowders in biological media with high concentration of proteins, polysaccharides, or nucleic acids.^91,92^ Yet, the CSW fluid has not a water-like entropy behavior, as all the other two-length scales isotropic potential, because it has no directional interactions.^85^ We compare the behavior of these two liquids with that of TIP4P/2005 water, in which, instead, the specific geometry of four charges induces the electrostatic interactions responsible for the HBs along preferred directions. HBs are responsible for the complex behavior of water (with the TIP4P/2005 performing well among the different classical models of water),^94^ playing a relevant role in the presence of graphene walls.^95^

The TIP4P/2005 water in a graphene slit-pore has free-energy extrema determining diffusion oscillations, with free-energy/diffusion minima for wall–wall distances fitting complete layers, down to one, and maxima at intermediate distances.^96^ In particular, the free-energy minimum for a monolayer originates from an increase of water disorder, despite the corresponding water internal energy increases. For the bilayer, instead, the free-energy minimum is dominated by a minimum in internal energy per water molecule with a larger order.^96^ The latter, with a full HB network, is the minimum with the largest mechanical stability,^96^ raising the question if it would be so also in a fluid without HBs.

## Results and Discussion

**Figure 1:**
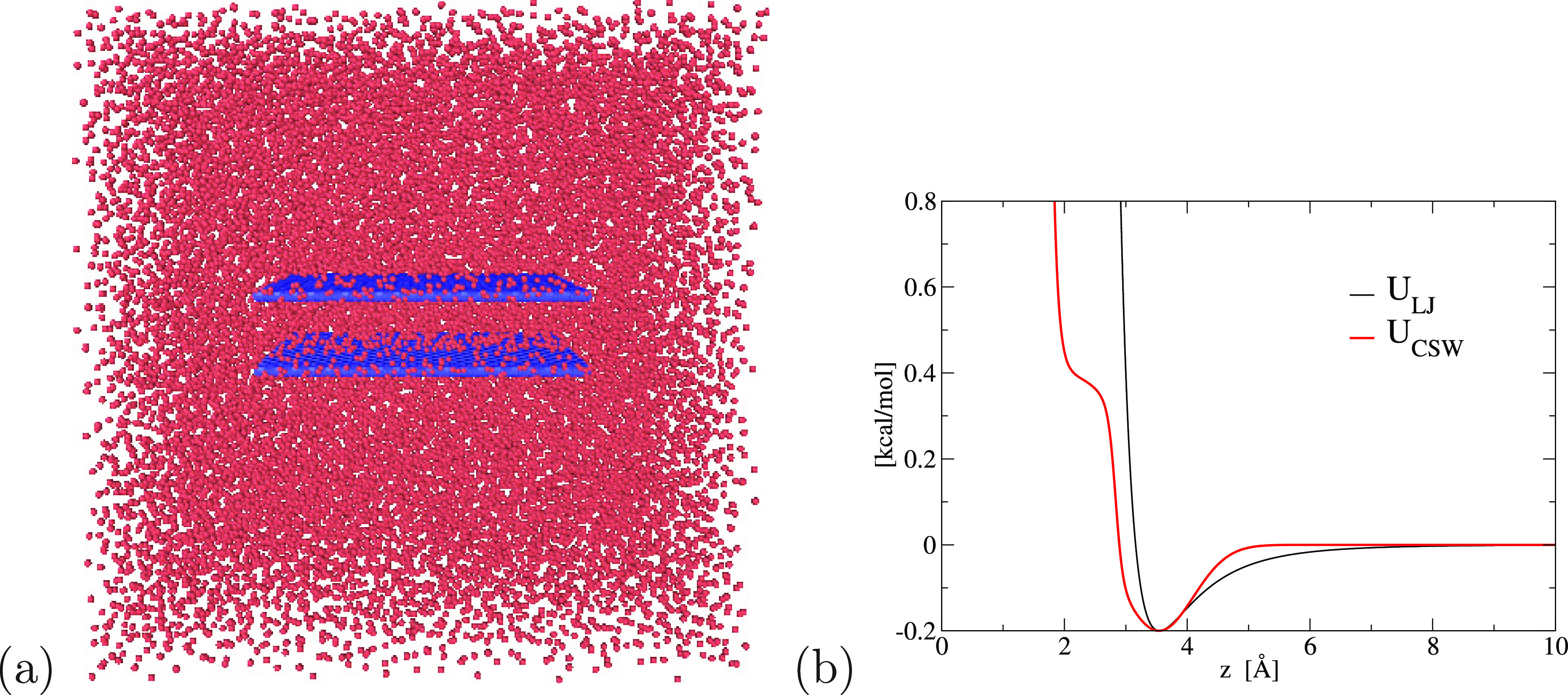
Simulation geometry and isotropic potentials. (a) Snapshot of the simulation box with *N*_tot_ = 25000 CSW particles at ρ = 0.036 Å^–3^ and *T* = 100 K, with a graphene slit-pore with width δ = 11 Å and area *A* ≃ 25 nm^2^. (b) LJ (black line) and CSW (red line) interparticle potentials for systems 1 and 2, respectively, as described in the text.

We perform MD simulations (see Methods section for details) of three different fluids surrounding a graphene slit-pore (Figure 1): (1) A simple fluid, described by a LJ potential; (2) an anomalous fluid, with water-like properties but different from water, modeled with the isotropic CSW potential; and (3) TIP4P/2005 water. For each fluid, we simulate a box, with periodic boundary conditions, with a slit-pore, centered at the origin of the reference system, made of two parallel graphene sheets of fixed area *A*, separated by a distance δ and positioned a *z*_*p*_±__ = ± δ/2. We consider nanoscopic slit-pores of width ranging from δ = 6.5 Å to δ = 17 Å, with 0.5 Å increments. To reduce the edge effects of the walls, we compute the properties only of the confined fluids with coordinates −*L*_*x*_^*s*^/2 < *x* < *L*_*x*_^*s*^/2, −*L*_*y*_^*s*^/2 < *y* < *L*_*y*_^*s*^/2, and *z*_*p*_–__ < *z* < *z*_*p*_+__, that is, within a central subvolume *V*^*s*^ ≡ *A*^*s*^ × δ, where *A*^*s*^ ≡ *L*_*x*_^*s*^*L*_*y*_^*s*^, with *L*_*x*_^*s*^ = *L*_*y*_^*s*^ = 30 Å for the isotropic potentials (1) and (2) and 15 Å for the TIP4P/2005 water (3).

### Structure

We first analyze how the confinement affects the structure of the isotropic liquids. As other confined liquids, the LJ and CSW fluids form layers parallel to the walls, displaying peaks in their density profiles ρ_*z*_(*z*) along the normal direction *z* (Figures S1 and S2). The number of layers increases with the distance 6.5 Å ≤ δ ≤ 17 Å between the plates, going from 1 to 4 for the LJ and from 1 to 5 for the CSW. The presence of two characteristic length scales in the CSW potential leads to the formation of complex patterns^83^ and structured peaks, especially at higher densities (not shown) that are absent in the LJ.

**Figure 2:**
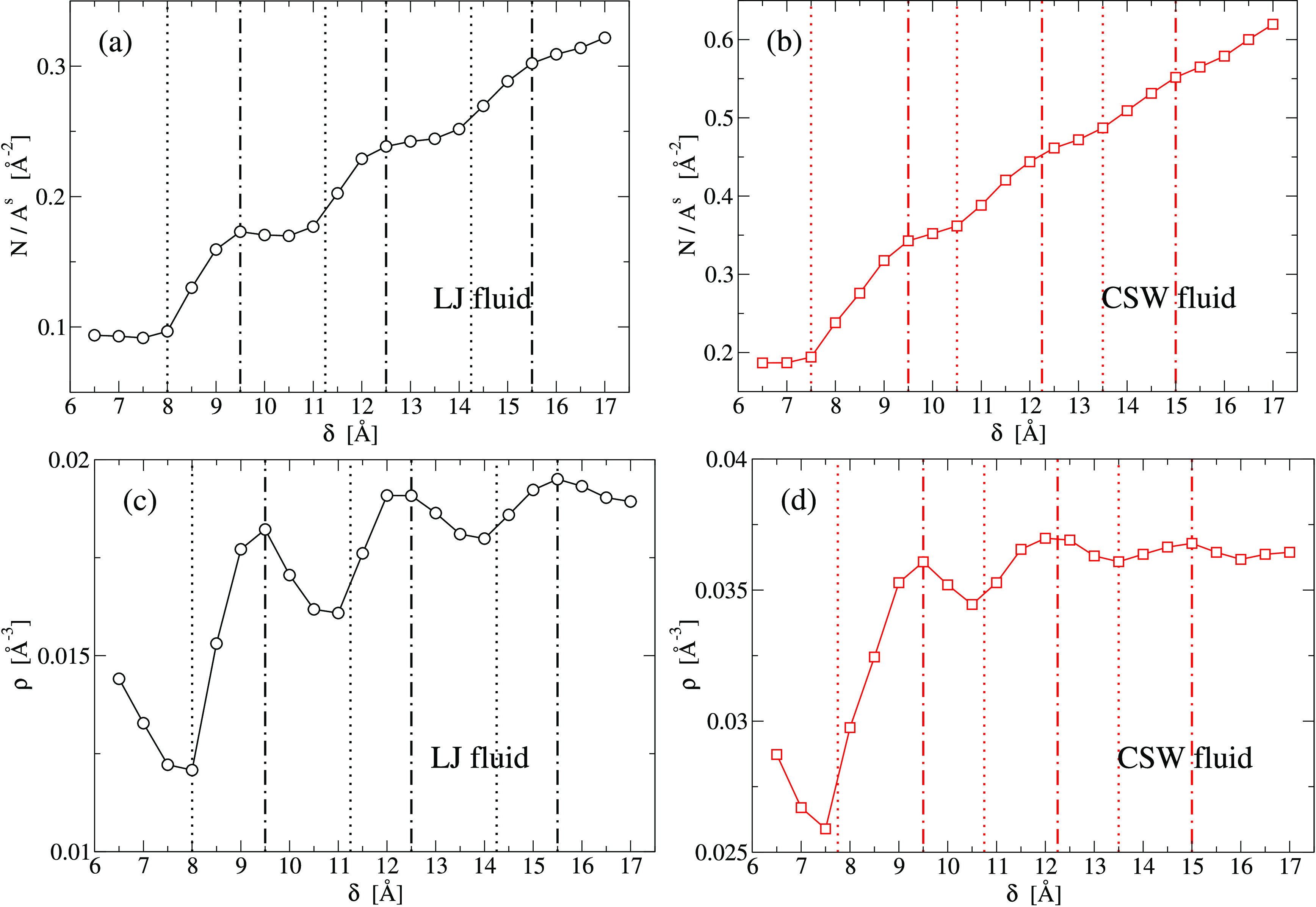
Slit-pore acceptance capacity, *N*^*s*^/*A*^*s*^, and mean density ρ for the confined isotropic fluids as a function of the plate separation δ. (a, b) The LJ parameters are ρ_LJ_ = 0.023 Å^–3^ and *T*_LJ_ = 100 K. (c, d) The CSW parameters are ρ_CSW_ = 0.036 Å^–3^ and *T*_CSW_ = 100 K. The (dotted and dot-dashed) vertical lines are defined in Figure 4 and mark approximately the extrema (maxima and minima, respectively) of the fluid diffusion coefficient *D*_∥_ in layers parallel to the plates.

We find that the slit-pore acceptance capacity, defined as the number of confined particles *N*^*s*^/*A*^*s*^ normalized by the subvolume area *A*^*s*^, for both fluids has a step-like behavior as a function of δ (Figure 2a,c). These steps resemble what has been found for water under similar confinement,^96,97^ and it is a result of the layering. Indeed, the comparison with Figures S1 and S2 shows that a step starts at values of δ where a new layer appears (*e*.*g*., for the LJ: δ ≃ 8 Å, 11.25 Å, 14.25 Å; for the CSW: δ ≃ 7.75 Å, 10.75 Å, 13.5 Å). The steps smoothen for larger δ as the confined fluid becomes less structured.

We can emphasize this behavior by analyzing how the mean density ρ of the fluid within the pore changes with δ (Figure 2c,d). It shows oscillations, approaching the bulk value for increasing δ. The mean density reaches minima (density minima inside the pore are larger than the gas density associated with the bulk liquids at the same thermodynamic conditions)^84^ at those separations where, for an increase of δ, the fluid starts a new layer and the particles are sucked inside the pore from the reservoir.

For intermediate separations, both liquids fill the layers up to reach maxima in ρ, corresponding to optimal plates distances (*e*.*g*., for the LJ: δ ≃ 9.5 Å, 12.5 Å, 15.5 Å; for the CSW: δ ≃ 9.5 Å, 12.25 Å, 15 Å) where the density profiles ρ_*z*_(*z*) display well-formed peaks, sharper and higher than those for slightly different δ (Figures S1 and S2). A further increase of δ, up to the value for a new layer, does not increase the acceptance capacity (plateaus in Figure 2a,b), leading to a new minimum in ρ. For the CSW fluid, the plateaus of the acceptance capacity and the oscillation in ρ are less pronounced and shifted toward smaller values of δ (Figure 2b,d) as a consequence of the interaction soft-core.

As we will discuss in the following sections, these steps and oscillations are associated with oscillatory behaviors in dynamics (vertical lines in Figure 2), hydration forces, and thermodynamics. In particular, the relation between structure and entropy can be emphasized by calculating the translational order parameter^98,99^ in each layer *i*, defined as
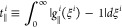
1where ξ^*i*^ ≡ *r*_∥_(ρ_∥_^*i*^)^1/3^ is the longitudinal distance *r*_∥_, parallel to the walls, in units of the mean interparticle separation (ρ_∥_^*i*^)^−1/3^, ρ_∥_^*i*^ is the fluid density in the layer *i*, and *g*_∥_^*i*^(ξ^*i*^) is the longitudinal radial distribution function. For an ideal gas, *g*_∥_(ξ) = 1, hence there is no translational order (*t*_∥_ = 0).

**Figure 3:**
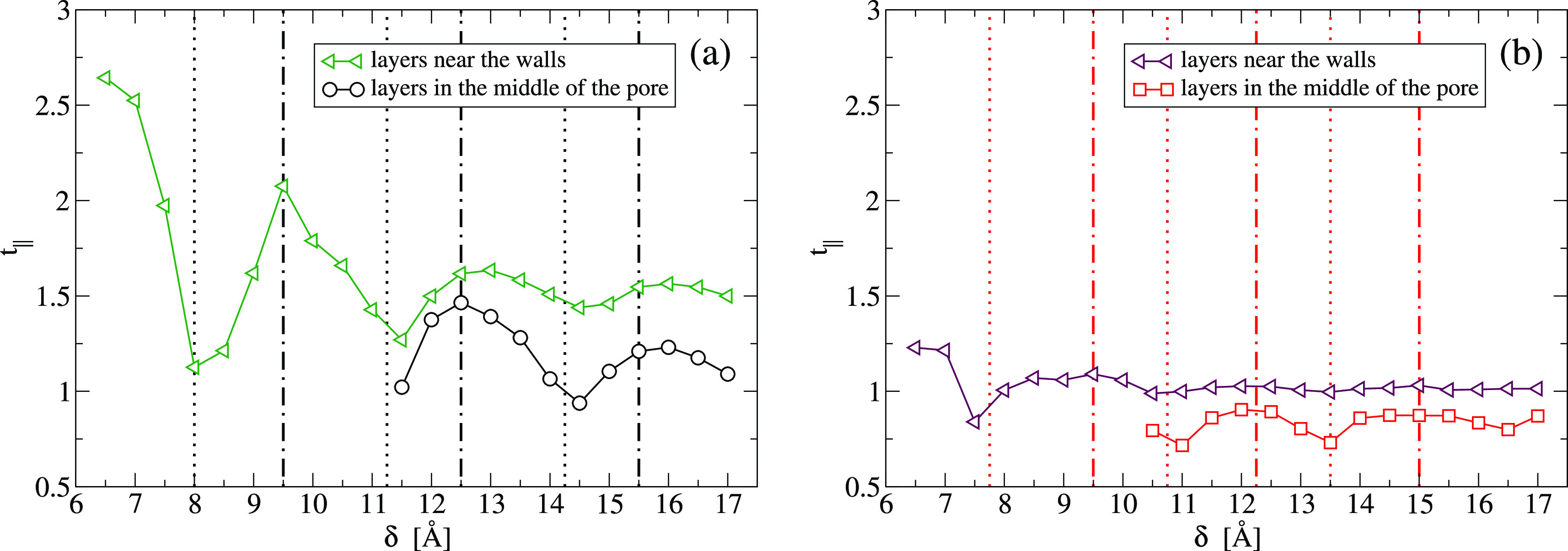
Longitudinal translational order parameter *t*_∥_ for each layer of confined isotropic fluids as a function of the plate separation δ. The parameter is calculated for the layers in contact with the walls (triangles) and, separately, for the other layers (circles for LJ, squares for CSW). The thermodynamic conditions for the LJ (a) and the CSW (b) and the vertical lines are the same as in Figure 2.

We calculate the parameter separately for the layers in contact with the walls and for the other layers (Figure 3), finding that both oscillate with δ and that the contact layers are always more ordered than the inner layers. However, they have maxima and minima at the same values of δ, showing that the plate separation can regulate the order in the whole confined fluid.

In particular, the layers are more ordered when the mean density ρ of the confined fluid is maximum, that is, when the δ is optimal for well-formed layers. The fluid order decreases when the mean density ρ is minimum, corresponding to the appearance/disappearance of a new layer.

For small δ, when the slit-pore contains only one or two layers of the fluid, *t*_∥_^*i*^ has larger oscillations, although for the CSW liquid, the variation is weaker, as a consequence of its soft-core. In general, the CSW is always less ordered than the LJ at the same plate separation δ. However, for both isotropic fluids, the structural oscillations, due to the layering, determine the translational order, hence the entropy, of the confined liquid and are correlated to its dynamics (vertical lines in Figure 3). In the next section, we show how we locate the vertical lines marking the extrema in the dynamics.

### Dynamics

**Figure 4:**
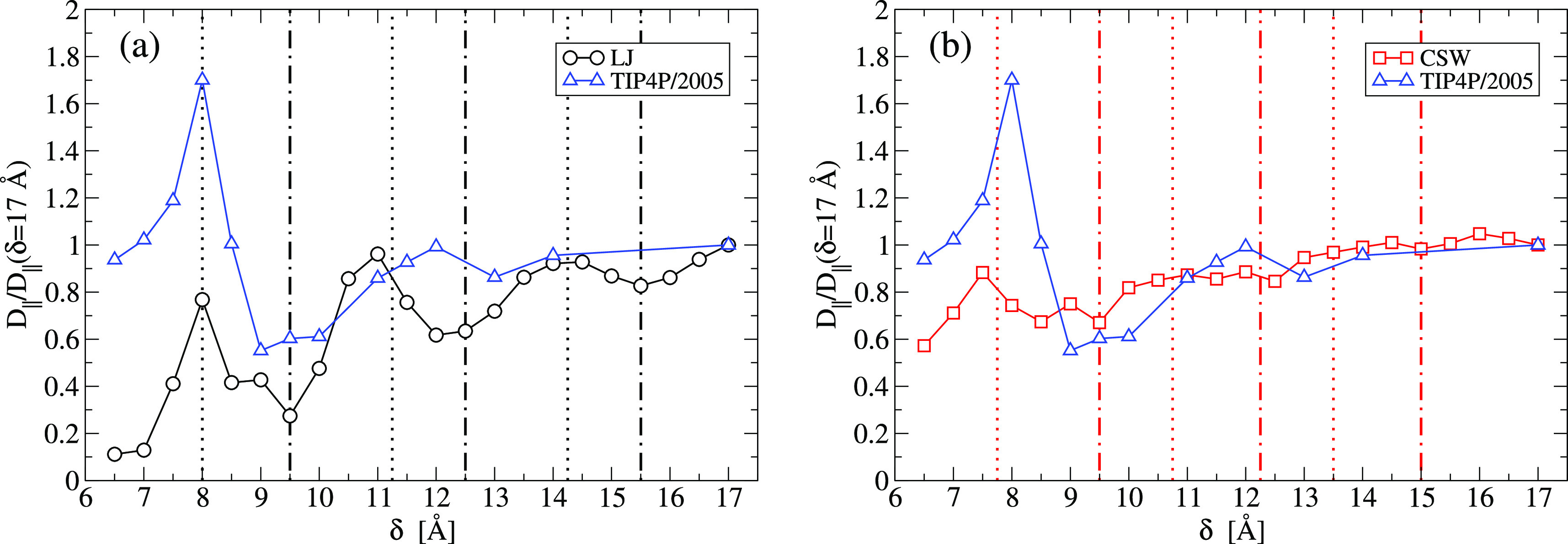
Longitudinal diffusion coefficient *D*_∥_, normalized to its large δ value, for the three fluids in a slit-pore, as a function of the plate separation δ. Comparison of the TIP4P/2005-water (blue triangles)^96^ with (a) the LJ (black circles) and (b) the CSW (red squares). In both panels, vertical lines mark, approximately, maxima (dotted lines) and minima (dot-dashed lines) for the isotropic fluid (see text). The value of *D*_∥_ at δ = 17 Å is ≃23 nm^2^/ns for both the LJ and the CSW and ≃1.9 nm^2^/ns for the TIP4P/2005-water.^96^

Next, we analyze how the confinement affects the thermal motion, in the direction parallel to the plates, by calculating the longitudinal diffusion coefficient, *D*_∥_, for our three prototypical liquids, as a function of the plates interdistance δ, with
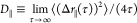
2where
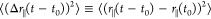
3is the longitudinal mean square displacement, with *r*_∥_ ≡ (*x*^2^ + *y*^2^)^1/2^, τ ≡ *t* – *t*_0_ is the time spent in the confined subregion *V*^*s*^ by a particle entering *V*^*s*^ at *t*_0_, and ⟨···⟩ is the average over 10 time intervals, each made of 10^4^ MD steps (Figure 4).

We observe that the three fluids share three properties:

(i) *D*_∥_ is not monotonic as a function of δ
(ii) *D*_∥_-oscillations are larger for smaller δ and
(iii) *D*_∥_ is not monotonic as a function of the average density ρ (Figure S4), indicating anomalous behavior

Because only the water^100^ and the CSW fluid^81^ can show anomalous diffusion in the bulk, while the LJ fluid cannot, we conclude that these three properties are not necessarily related to the bulk anomalies.

The property (iii) resembles recent results for other confined anomalous-fluid models where it was attributed to the competition of two interaction length-scales,^10,101^ the appearance of amorphous phases,^101^ or the re-entrance of the melting line.^102^ However, here we find it also for the simple fluid without competing length-scales, amorphous phases, or re-entrant melting, showing that the presence of confinement is enough to get the property (iii), as well as the (i) and (ii), in the three fluids.

Nevertheless, there are relevant differences among the three cases.

(iv) For both isotropic (LJ and CSW) fluids, *D*_∥_ oscillates but is always smaller than its bulk value, with a decreasing trend for decreasing δ. In the case of water, instead, the fastest diffusion is reached at δ ≈ 8 Å, between one and two confined layers.^96^
(v) Although both the isotropic fluids have, for the selected state-point, a diffusion coefficient *D*_∥_ ≃ 23 nm^2^/ns for δ = 17 Å (≃10 times larger than the value for water), the oscillations of *D*_∥_ in the three fluids are quite different: ≈90% for LJ, ≈40% for CSW, and ≈70% for water.
(vi) For subnm confinement (δ < 10 Å), the minima and maxima of the *D*_∥_ oscillations are approximately located at the same separations for the three liquids. However, the oscillations mismatch for δ > 10 Å, being opposite at δ ≈ 12.5 Å, especially comparing LJ and water.

The subnm correspondence is better between LJ and water because the size of the LJ particles is equal to that of the LJ-component of the water model, while the CSW soft-core reduces the effective size of the particles and smoothens out the effect. The matching of the oscillations for δ < 10 Å confirms^96^ that the steric hindrance (layering) has a major role in determining the dynamics under confinement of a simple liquid as well as an anomalous liquid. However, this mechanism is no longer enough to rationalize the behavior for larger confinement, as emphasized by the mismatch for δ > 10 Å and the differences highlighted in (iv) and (v). This observation calls for an alternative explanation for the peculiar dynamics of confined water. As we will show in the following, it is related to the specific properties of the water HBs.

### Thermodynamics

#### Hydration Pressure

**Figure 5:**
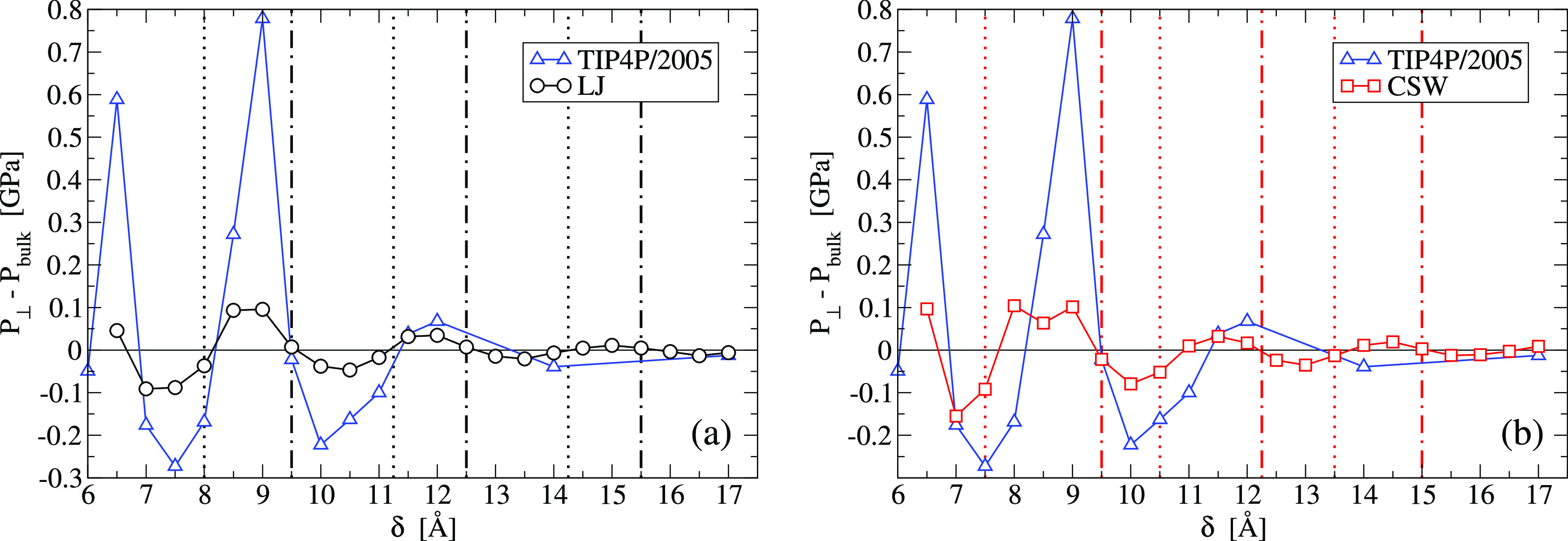
Hydration pressure, *P*_hydr_, for the confined fluids as a function of the plate separation δ. Comparison of the TIP4P/2005-water (blue triangles)^96^ with (a) the LJ (black circles) and (b) the CSW (red squares). In both panels, the (dotted and dot-dashed) vertical lines are those indicated in Figure 4, marking approximately the extrema (maxima and minima, respectively) of *D*_∥_ for the isotropic fluids. We observe that all the lines in panels (a) and (b) approximately cross the zeros of *P*_hydr_ for the LJ (a) and the CSW fluids (b), respectively.

To better understand the differences between the three confined fluids, we calculate the hydration pressure, *P*_hydr_ ≡ *P*_⊥_ – *P*_bulk_, as a function of δ. Here, *P*_⊥_ is the normal pressure that the confined fluid exerts on the plates, and *P*_bulk_ is the bulk pressure (our total system is large enough with respect to the confined region to keep the bulk pressure approximately constant even when we fix the total volume, as in the CSW fluid and TIP4P/2005 water, instead of *P*_bulk_. We verify that the parallel pressure inside the pore is equal to *P*_bulk_, as expected at equilibrium^83,103^) (Figure 5).

Over the entire range of δ explored here, we find that *P*_hydr_ oscillates for the three fluids and approaches zero at δ = 17 Å. Hence, at large plates separation, the confined fluids behave as in the bulk. When *P*_hydr_ > 0, there is an effective repulsion between the plates, while when *P*_hydr_ < 0, there is a fluid-mediated attraction. In both cases, the walls are kept fixed in their position by our simulation constraints. The constraint is not necessary when, instead, the walls are at equilibrium, with *P*_hydr_ = 0.

We observe that, for LJ and CSW fluids, the equilibrium δ-values, at which *P*_hydr_ = 0, coincide, within our numerical precision, with the extrema of *D*_∥_. Hence, the system is in equilibrium not only when the thermal diffusion is minimal but also when it is maximal. This suggests that the two equilibrium positions have a very different origin, as already observed in the case of water.^96^

In particular, if δ_1_^max *D*_∥_^ and δ_1_^min *D*_∥_^ are the shortest distances for a maximum and a minimum *D*_∥_, respectively, displacing the pore-size from δ_1_^min *D*_∥_^ induces a change in pressure that tends to restore the wall-to-wall distance. Hence, δ_1_^min *D*_∥_^ corresponds to a distance of stable mechanical equilibrium. The opposite occurs around δ_1_^max *D*_∥_^, hence, it corresponds to a distance of unstable mechanical equilibrium.

By decreasing δ from δ_1_^min *D*_∥_^ to δ_1_^max *D*_∥_^, *P*_hydr_ increases up to a maximum and, at intermediate distances, decreases toward *P*_hydr_ = 0. Hence, squeezing the fluid toward δ_1_^max *D*_∥_^ implies a speedup of the thermal diffusion and a work against the effective wall–wall repulsion.

At δ_1_^max *D*_∥_^ the fluid has maximum diffusion at unstable mechanical equilibrium, *P*_hydr_ = 0. Any further squeezing induces an attraction, *P*_hydr_ < 0, between the walls. In this case, the work to reduce δ is done by the fluid-mediated wall–wall attraction and slows down the thermal parallel diffusion.

Between the two equilibrium values δ_1_^max *D*_∥_^ and δ_1_^min *D*_∥_^, *P*_hydr_ for LJ and TIP4P/2005 liquids displays a single peak, while the CSW fluid has two close peaks. This difference can be understood as a signature of the two competing length scales of the CSW potential. Similar considerations hold for all δ_*i*_^max *D*_∥_^ and δ_*i*_^min *D*_∥_^, although we find only simple maxima of *P*_hydr_ for the CSW.

We observe that the water *P*_hydr_ has its largest maximum (repulsion) around δ ≃ 9 Å, corresponding to a confined bilayer, with a smaller maximum for the monolayer at δ ≃ 6.5 Å and the trilayer at δ ≃ 12 Å. For the isotropic fluids, instead, the maxima in *P*_hydr_ for the bilayer and the monolayer are approximately equal and larger than those for more layers, at least within our resolution. This observation suggests that the work to approach the walls at a bilayer is larger than at a monolayer for water, while is it approximately the same for the isotropic fluids. This is consistent with the result showing that the bilayer is more stable than the monolayer for water^96^ and suggests that it is not for the isotropic fluids. To deepen this understanding, we calculate, and compare, the free energy of the confined fluids in the next section.

#### Free Energy

**Figure 6:**
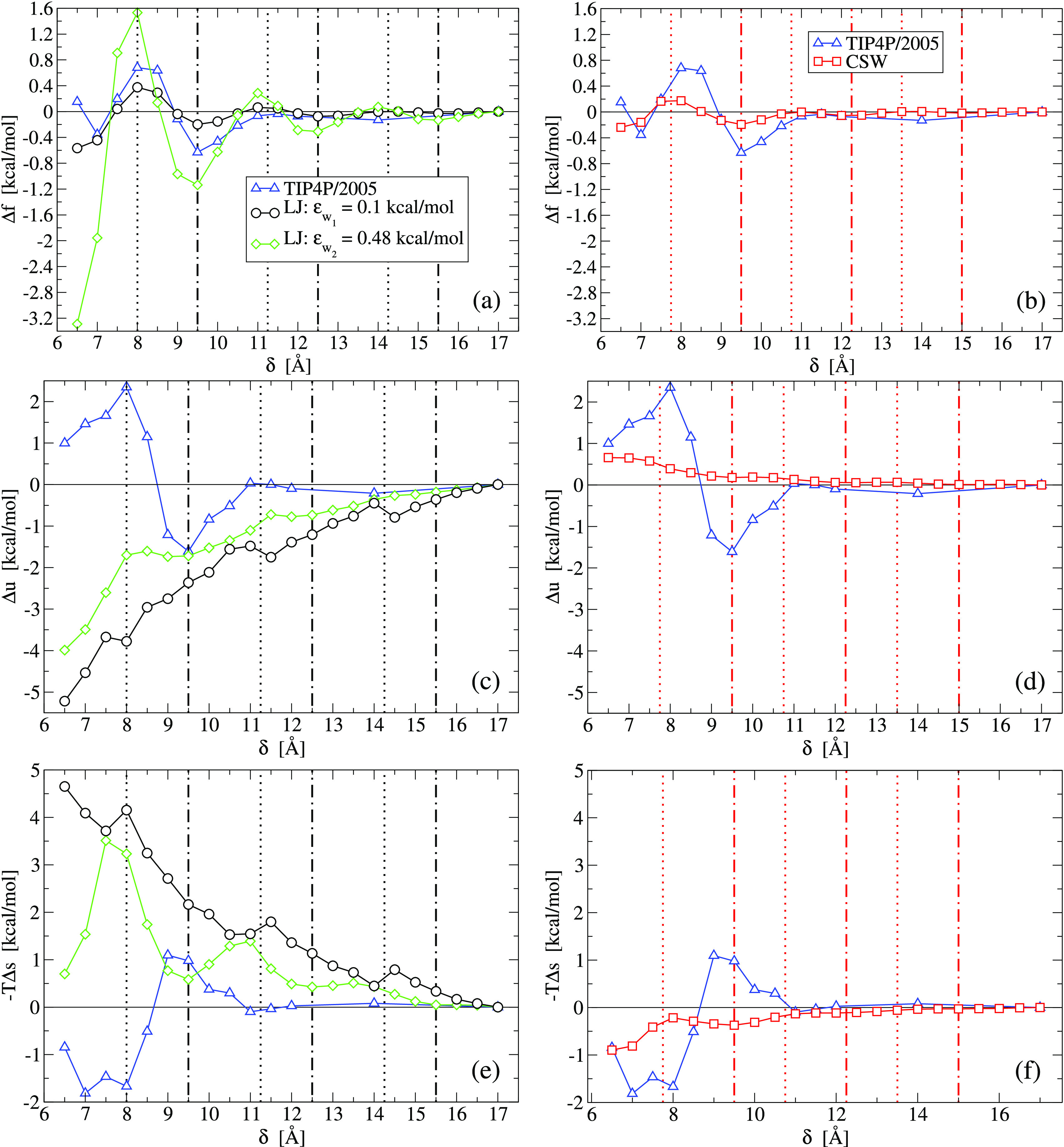
Variation of (a, b) the free-energy density, Δ*f*, (c, d) the internal energy density, Δ*u*, and (e, f) the entropy density, −*T*Δ*s*, for the confined fluids when the plate separation changes from δ_0_ = 17 Å to δ. Comparison of the TIP4P/2005-water (blue triangles)^96^ with (left panels) the LJ with fluid–wall interaction energy ϵ_w_1__ = 0.10 kcal/mol (black circles) and ϵ_w_2__ = 0.48 kcal/mol (green diamonds) and (right panels) the CSW (red squares). In all the panels, vertical lines are as in Figure 4, marking maxima (dotted lines) and minima (dot-dashed lines) in *D*_∥_. We find that the lines of *D*_∥_ maxima and minima coincide with the Δ*f* maxima and minima, respectively, for the LJ (left panels) and the CSW fluids (right panels).

Following refs (96, 104, and 105), we compute the macroscopic free-energy variation per particle, Δ*f*, as the macroscopic work done against the hydration forces to change the pore size from δ_0_ to δ, over the *N*^*s*^ molecules, confined within the pore subvolume of area *A*^*s*^, as
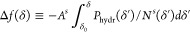
4We numerically calculate Δ*f*(δ) from the largest plates separation δ_0_ = 17 Å to a generic value δ, by setting in eq 4*d*δ = 0.5 Å as our minimal incremental value of δ (Figure 6a,b).

Furthermore, we calculate the variation of the internal energy per particle of the confined fluid, *Δu*(δ) ≡ *U*(δ) /*N*^*s*^(δ) – *U*(δ_0_)/*N*^*s*^ (δ_0_), where *U*(δ) is the internal energy of the confined fluid at plates separation δ (Figure 6c,d). Finally, we estimate the variation of the entropy per particle of the confined fluid as −*T*Δ*s*(δ) = Δ*f*(δ) – Δ*u*(δ) (Figure 6e,f).

We find that the LJ and the CSW fluid present oscillations of Δ*f* in phase with those for the TIP4P/2005 water (apart from the oscillation around δ ≃ 14 Å that for water is not observed, possibly, for lack of resolution). Furthermore, the CSW liquid and the LJ with weaker fluid-wall interaction (LJ_w_, with ϵ_w_1__ = 0.10 kcal/mol) are qualitatively very similar, with smoother oscillations for the CSW due to its pronounced soft core, as already observed for *D*_∥_ (Figure 4). Nevertheless, we observe important differences between the two isotropic fluids and the water.

First, the internal energy, Δ*u*, and entropy, −*T*Δ*s* for the isotropic fluids oscillate but never change sign, while they do for water. In particular, for the LJ_w_ the Δ*u* is always negative and the −*T*Δ*s* is always positive, while for the CSW the signs are inverted. Nevertheless, the two contributions sum up in a similar Δ*f* for both isotropic fluids.

Second, for small pores the Δ*f* for the isotropic fluids has deeper minima. Hence, their stability increases for smaller pore sizes and is maximum for the monolayer. Instead, for water the deeper minimum of Δ*f* is for the bilayer.^96^

Third, for the LJ_w_, the entropy variation −*T*Δ*s*(δ) is positive and on average increases for decreasing δ. Hence, the structural order of these confined liquids increases when the pore size decreases, consistent with our calculations of the longitudinal translational order *t*_∥_ (Figure 3a). For the CSW, −*T*Δ*s*(δ) is negative and *t*_∥_ is almost constant (Figure 3b), suggesting that the translational order has a minor effect in the calculations of −*T*Δ*s*(δ) for the confined CSW. For water, instead, −*T*Δ*s*(δ) is negative for δ ≲ 8.7 Å, for a confined monolayer, and positive for a confined bilayer, around δ = 9.5 Å.^96^ Hence, a confined water monolayer is less ordered than bulk water, while a water bilayer maximizes the structural order.

Hence, comparing the three models, we can state that the more stable free energy minimum for water is the bilayer, and it is energy driven and more structured than bulk. The monolayer of water is less stable, and it is entropy driven. For the isotropic fluids, the more stable free energy minimum is for the monolayer. For the LJ_w_ ,it is energy driven, while for the CSW, it is entropy driven.

#### Dependence of the Free Energy on Fluid–Wall Interaction

Qualitative differences in the excess free energy between (SPC/E) water and a LJ fluid have been found also with density functional theory as a function of the fluid–wall interaction, although between face centered cubic (fcc)-structured slabs.^106^ Hence, to understand how our results depend on the fluid-wall interaction, we consider a LJ liquid with a strong wall-attraction energy (LJ_s_), with ϵ_w_2__ = 0.48 kcal/mol (green diamonds in left panels of Figure 6).

We find that the free energy oscillation for the LJ_s_ are stronger than for the LJ_w_, but the minima and maxima occurs, approximately, at the same pore sizes δ. In particular, the entropy oscillations of the LJ_s_ are large, showing that the stronger fluid–wall interaction has a larger structural effect with respect to the LJ_w_ case.

**Figure 7:**
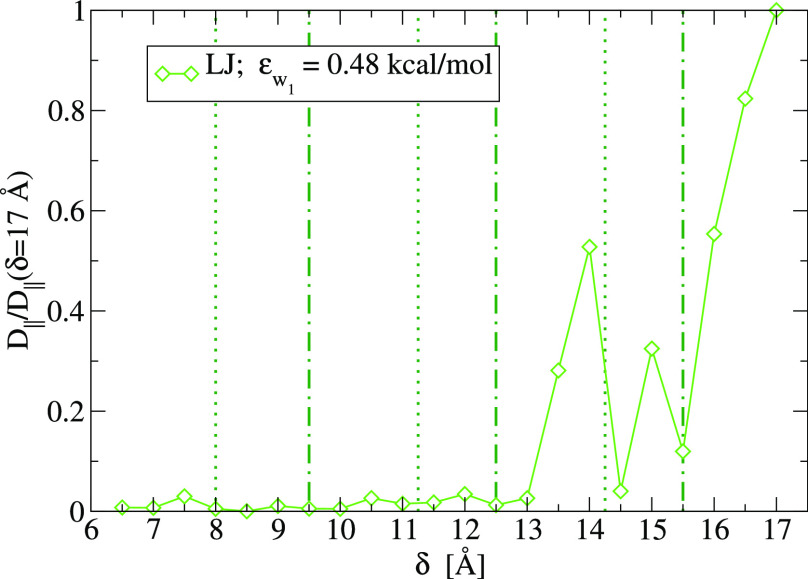
Longitudinal diffusion coefficient *D*_∥_, normalized to its large δ value, for the LJ_s_ with strong fluid–wall interaction ϵ_*w*_2__ = 0.48 kcal/mol in a slit-pore, as a function of the plate separation δ. The vertical lines mark approximately maxima (dotted lines) and minima (dot-dashed lines) of Δ*f* for the LJ_s_ fluid (Figure 6). The value of *D*_∥_ at δ = 17 Å is ≃23 nm^2^/ns.

This is confirmed when we calculate the longitudinal diffusion coefficient *D*_∥_ for the LJ_s_ (Figure 7). We find that, at variance with the LJ_w_ case (Figure 4a), the LJ_s_ freezes for δ ≤ 13 Å. The parallel diffusivity inside the pore goes to zero when all the fluid layers are frozen, in a distorted triangular lattice, which happens when the peaks of the density profile are completely formed and there are no particles in between (Figure S4).

Crystallization and dynamic freezing have been found also for water confined into a graphene slit-pore when TIP4P/2005-water is at high pressure (*P* = 400 bar) and a temperature (*T* = 275 K) below the one considered here.^107^ However, it occurs for a bilayer that, as seen above, is the more stable configuration for confined water. Under these conditions, TIP4P/2005-water crystallizes into a hexagonal bilayer^107^ at a temperature that is much above the bulk melting temperature^108^ (*T*_m_(*P* = 400 bar) < *T*_m_(*P* = 1 bar) = 249.5 ± 0.1 K).^109^ As a consequence of the large bilayer stability, the confined crystal undergoes re-entrant melting when the pore size allows only a water monolayer.^107^

These results show that a strong fluid–wall interaction can induce crystallization in both confined LJ and water; however, they do not rationalize the subnm speed-up and the bilayer strong stability that we find in water. Hence, these properties are specific of confined water, possibly related to its HBs. Indeed, the HB network and its specific geometry are held responsible for the crystallization of subnm confined water into bilayer ices at ambient conditions in experiments^16,110,111^ and simulations^30,112−117^ and its re-entrant melting by changing the slit-pore size.^107,112,113^ To understand better how it relates to the subnm speed-up and the bilayer strong-stability, we analyze the water HB network in the detail in the next section.

#### The Confined Water HB Network

**Figure 8:**
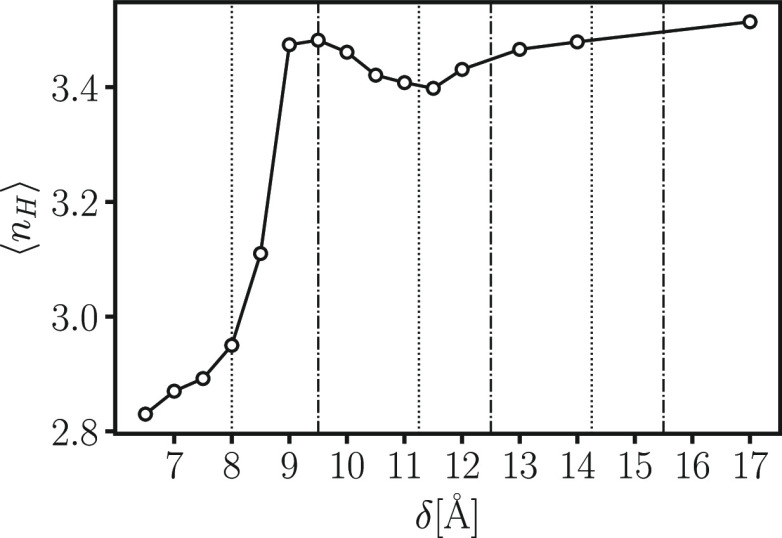
Average number of HBs per molecule, ⟨*n*_HB_⟩, for the water in the confined subvolume, *V*^*s*^, as a function of the pore size δ. Vertical lines are defined as in Figure 4, approximately marking maxima (dotted lines) and minima (dot-dashed lines) in *D*_∥_ and coinciding with Δ*f* maxima and minima, respectively, for the water in Figure 6 (left panels).

First we calculate the average number of HBs per molecule, ⟨*n*_HB_⟩, for the water in the confined subvolume, *V*^*s*^, as a function of the pore size δ (Figure 8) (vertical lines in the figure are defined for the LJ oscillations, but, as discussed in the text, they approximate well the water oscillations). We find that ⟨*n*_HB_⟩ is almost as large as in bulk for the bilayer, where the free energy and *D*_∥_ have their absolute minima. For other values of δ, ⟨*n*_HB_⟩ is smaller, with a local minimum at δ ≃ 11.5 Å, where both Δ*f* and *D*_∥_ have local maxima.

These observations suggest that both diffusion and free energy are dominated, in the range of δ, by the average number of HBs. However, for δ < 9 Å, the analysis is less intuitive. Indeed, the maximum in *D*_∥_ at δ ≃ 8 Å does not correspond to a minimum in ⟨*n*_HB_⟩ (Figure 8). Counterintuitively, for δ < 8 Å, both ⟨*n*_HB_⟩ and *D*_∥_ decrease.

**Figure 9:**
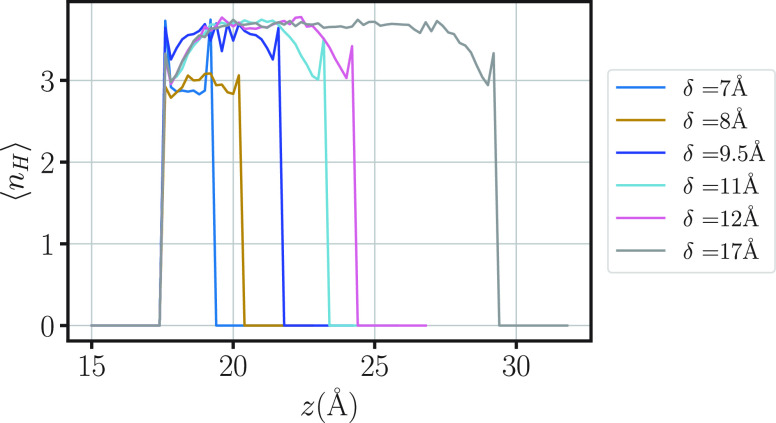
HB profile as a function of the water molecule position along the direction *z* perpendicular to the slit-pore walls, for 7 ≤ δ/Å ≤ 17. For δ ≥ 9.5 Å, the profile saturates in its center to the bulk value ⟨*n*_HB_⟩ ≃ 3.5, while it is less near the walls. For δ ≤ 8 Å, it is ⟨*n*_HB_⟩ ≃ 3 in the center and ⟨*n*_HB_⟩ ≃ 3.5 near the walls. Colors for each δ are indicated in the legend. For sake of comparison, for each δ, the first peak of the profile is shifted at *z*_0_ = 17.5 Å. The distance from the first peak and the nearest wall can be estimated from Figure 5 of ref (96)—very similar to Figures S1 and S2 for the LJ—and changes with δ. The thickness of the HB profile changes with the thickness of the density profile in the same figures.

This is the range of δ values where the confined-water free-energy is dominated by its entropy. In particular, its −*T*Δ*s* has a (structured) minimum for 7 ≲ δ/Å ≲ 8 (Figure 6e). Although not evident from the averaged ⟨*n*_HB_⟩, our detailed analysis shows that, for these values of δ, the HB profile is quite different from the cases at larger δ. We find (Figure 9) that the HB profile for δ > 8 Å (with two or more layers) saturates in its center to a bulk-like value within ≃4.5 Å from the graphene wall. For δ ≤ 8 Å, there is not enough space in the pore to allow the water molecules to arrange in such a saturated network. As a consequence, away from the wall, the profile reaches a local value of ⟨*n*_HB_⟩ ≃ 3, indicating a less connected network.

**Figure 10:**
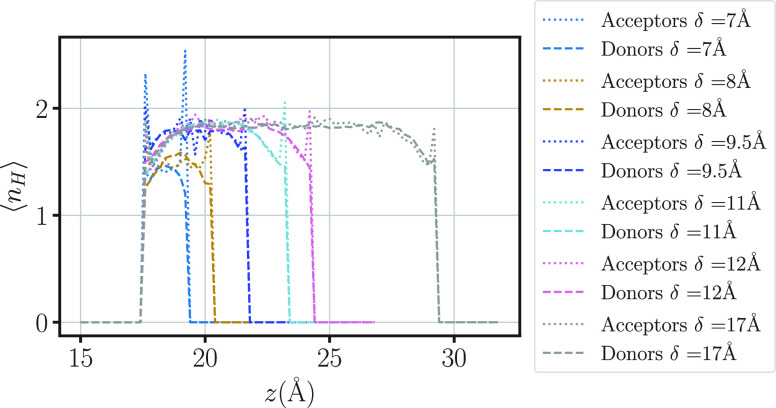
Profiles of HB acceptors (dotted lines) and donors (dashed lines) as a function of the water molecule position along the direction *z* perpendicular to the slit-pore walls, for 7 ≤ δ/Å ≤ 17. Colors are as in Figure 9.

In particular, we calculate the profiles of donors and acceptors for the HBs as a function of *z*, for each δ (Figure 10). We find that for δ = 7 Å (monolayer), the majority of the water molecules have their hydrogens pointing toward the center of the pore, away from the hydrophobic walls, as one would expect. This asymmetry between the donors and acceptors profiles smoothens for δ > 8 Å. The strong asymmetry for δ ≤ 8 Å indicates that the HB network is hindered by the hydrophobic wall, facilitating the breaking of the cooperative rearranging regions and the diffusion in confined water.^118^ This observation is consistent with the larger entropy of the confined water monolayer with respect to the cases with more, well-formed layers (Figure 6e).

## Conclusions

We compare structure, dynamics, and thermodynamics of water confined in a graphene slit-pore with two isotropic liquids, a simple liquid (LJ) and an anomalous liquid (CSW), under similar conditions. We find that below ≃1 nm, where only two or one layer can be accommodated, confined water is different for, at least, the following reasons.

(i) Water goes from very large to very small order, changing the pore size from 0.95 to 0.80 nm, when compared with the bulk. The considered isotropic liquids, instead, have a structural order that, although oscillating, increases in its maxima for decreasing pore size.
(ii) Water goes from less to more diffusive than bulk changing the pore size from 0.95 to 0.80 nm, with a maximum at 0.8 nm. The isotropic liquids, instead, have a thermal diffusion oscillating with the pore size, but with an overall decreasing diffusion coefficient for decreasing pore size.
(iii) Water has its maximum stability for the double layer at 0.95 nm, where it saturates its HB network. The monolayer at ≃0.7 nm is less stable and more disordered, with its HB network hindered by the hydrophobic graphene walls. For the isotropic liquids, instead, a monolayer is more stable than two or more confined layers. While for the simple LJ, the internal energy of the confined liquid is the leading contribution to the stability, and for the anomalous liquid, CSW, it is the entropy, resembling more water.

Our analysis clarifies that these differences are all due to the water HB network. Therefore, the layering alone is not able to rationalize the properties of water under subnm confinement, not even if a stronger interaction with the walls is considered. We find that strong LJ–wall interaction leads to freezing and crystallization at subnm pore-size, with an effect similar to a decrease of temperature for confined water^96^ and opposite to increase in diffusion or disorder.

Nevertheless, it is intriguing to observe that the differences with isotropic liquids fade out for pore with more than two layers (>1 nm). This is especially true for the CSW anomalous liquid that, in its bulk version, has some water-like properties,^81,83,84^ although not the entropy balance observed in water.^82,85^ However, the differences are emphasized for monolayers and bilayers that are common in biology and nanofluidics. For example, in water-soluble macrocyclic hosts, including aqueous synthetic receptors with an ultrahigh affinity binding for molecular recognition and sensing, materials chemistry, and drug delivery, the unsaturation of HBs under heavy confinement alters dramatically the properties of water to such an extent that the system employs cavitation to optimize its energy.^119^ Our results for monolayers allude that cavitation cannot be excluded *a priori*, especially, for δ < 6.5 Å (not presented here). Indeed, our inspection of preliminary snapshots at δ < 6.5 Å suggests the occurrence of cavitation, consistent with previous results for SPC/E-water confined between hydrophobic atomically flat walls.^120^ Hence, although we do not observe cavitation for the cases presented here, further study is needed to explore the relevance of cavitation in hydrophobic nanopores smaller than those in the present work. In conclusion, our results help to better understand how biology takes advantage of the peculiar properties of water and how nanotechnology could mimic, in this respect, Mother Nature.

## Methods

### Confined LJ Fluid

We simulate particles interacting *via* a LJ potential (Figure 1b):
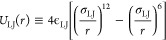
5with σ_LJ_ = 3.16 Å and ϵ_LJ_ = 0.2 kcal/mol. These parameters are chosen in such a way to compare with the LJ contribution of the TIP4P/2005 potential (with same size and 0.185 kcal/mol as LJ energy).^121^ In order to reduce the computational cost, we impose a cutoff for the interaction potential at a distance *r*_c_ = 10 Å.

The slit-pore is composed of two parallel graphene sheets. Each sheet is a honeycomb lattice made of *N*_G_ = 960 frozen particles, with interparticle distance 1.42 Å, lateral sizes *L*_*x*_ = 49 Å and *L*_*y*_ = 51 Å, and an area *A* ≡ *L*_*x*_ × *L*_*y*_≃ 25 nm^2^. The graphene particles of the walls interact with the fluid particles through a LJ potential, as in eq 5,^122^ with size σ_w_ = 3.26 Å and energy ϵ_w_1__ = 0.1 kcal/mol (case 1, weaker than the fluid–fluid interaction) or ϵ_w_2__ = 0.48 kcal/mol (case 2, stronger than the fluid–fluid interaction). The two choices, ϵ_w_1__ and ϵ_w_2__, allow us to study the effects of the fluid-wall interaction strength. In particular, we chose ϵ_w_2__ = 2.4 × ϵ_LJ_ to compare our results with those by Gao *et al*.^104,105^

We perform NPT simulations at constant number *N*_tot_ = 25000 of LJ particles, constant temperature *T* = 100 K, and constant bulk pressure *P*_bulk_ = 1 atm, leaving the box volume, *V* ≡ *L*_*x*_^box^ × *L*_*y*_^box^ × *L*_*z*_^box^ with *L*_*x*_^box^ = *L*_*y*_^box^, free to change (at this state point, corresponding to a bulk number density ρ_bulk_ ≃ 0.023 Å^–3^, *i*.*e*., a reduced density ρ_bulk_^*^ ≡ ρ_bulk_σ_LJ_^3^ ≃ 0.73, the bulk is liquid and the confined region is filled with fluid).

We simulate the system with LAMMPS, adopting the Nose–Hoover thermostat and barostat,^123^ with relaxation time 10^2^ and 10^3^ MD steps, respectively, and with 10^5^ MD steps of relaxation, enough to reach equilibrium in the bulk and within the confined subregion. Next we compute the observables for 10^5^ more MD steps, recording each quantity every 10^3^ MD steps.

### Confined CSW Fluid

We describe the anomalous fluid with the CSW potential (Figure 1b):^80−84^
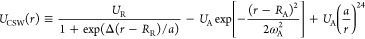
6where *a* is the diameter of the particles, *R*_A_ and *R*_R_ are the distance of the attractive minimum and the repulsive radius, respectively, *U*_A_ and *U*_R_ are the energies of the attractive well and the repulsive shoulder, respectively, ω_A_^2^ is the variance of the Gaussian centered in *R*_A_, and Δ is the parameter that controls the slope between the shoulder and the well at *R*_R_. We choose the CSW parameters in such a way that the resulting potential compares at best with LJ potential (Figure 1b): *U*_A_ = 0.2 kcal/mol, *a* = 1.77 Å, *R*_A_ = 2*a* ≃ 3.54 Å, *U*_R_/*U*_A_ = 2, *R*_R_/*a* = 1.6, (ω_A_/*a*)^2^ = 0.1, Δ = 30, and a cutoff at a distance *r*_c_ = 10 Å.

We adopt the same slit-pore as for the LJ fluid with the weak fluid-wall interaction, ϵ_w_1__ = 0.1 kcal/mol, simulating the system with LAMMPS and Nose–Hoover thermostat,^123^ with the same equilibration and production statistics as for the LJ. We perform the simulations at constant number *N*_tot_ = 25000 of CSW particles, constant temperature *T*, and constant box volume *V*, leaving the bulk pressure *P*_bulk_ free to change (simulations at constant *P*_bulk_ for the CSW fluid at the same *T*, same *P*_bulk_, and same *N*_tot_ as for the LJ fluid would require a much larger box for the CSW than the LJ in order to get a comparable number of particles inside the subvolume *V*^*s*^). We consider different values of temperature, *T*/K = 60, 80, 100, and we vary *L*_*x*_^box^ = *L*_*y*_^box^, changing the box section parallel to the slit-pore plates, to control the bulk number density as ρ_bulk_/Å^–3^ = 0.027, 0.036, 0.045, 0.054, that is, reduced densities ρ_bulk_^*^ ≡ ρ_bulk_*a*^3^ = 0.15, 0.2, 0.25, 0.30, all corresponding to the bulk liquid phase^82^ (Figures S1 and S2). We focus on the state point at ρ_bulk_ = 0.036 Å^–3^ and *T* = 100 K because it shows a dynamics comparable to the confined LJ, as discussed in the main text.

### Confined TIP4P/2005 Water

For the confined TIP4P/2005 water,^121^ we use the data and the parameters as described in ref (96). Specifically, the system has *N*_tot_ = 2796 water molecules in a box with constant volume *V* = 4.2 × 4.2 × 5.1 nm^3^ and constant *T* = 300 K, corresponding to *P*_bulk_ = 400 atm and a density ρ_bulk_ ≈ 1 g/cm^3^, that is, a number density 0.033 Å^–3^. The graphene slit-pore has two 24.6 Å × 25.5 Å rigid plates, and water–carbon interactions modeled as LJ potential as in the CHARMM27 force field, adopting the Lorentz–Berthelot rules, a cut off of the van der Waals interactions at 12 Å, a smooth switching function starting at 10 Å, and the particle mesh Ewald method,^124^ with a grid space of ≈1 Å, for the calculation of the long-range electrostatic forces. We integrate the equations of motion by using GROMACS^125^ with a 1 fs time-step and update the electrostatic interactions every 2 fs. We employ the Berendsen thermostat with relaxation time 0.5 ps (see ref (96) for a discussion on thermostats). Before collecting data for the analysis, we equilibrate the system for 5 ns. Then, we select data every 10 ps for the next 50 ns and every 0.1 ps for the next 8 ns. As for the fluids (1) and (2), also in this case, the observables are calculated in a confined subvolume *V*^*s*^, at constant *T*, and constant chemical potential μ. Further details are given in ref (96).

## Supporting Information Available

Density profiles of the LJ and the isotropic core-softened potential model; normal pressure and parallel diffusion coefficient of the isotropic core-softened potential model; snapshots of the LJ liquid with strong fluid–wall interaction (PDF)
