# Supplementary material for: Nanoconfined Fluids: Uniqueness of Water Compared to Other Liquids
Source: ACS Nano. 2021 Nov 22;15(12):19864–76. doi: 10.1021/acsnano.1c07381 (PMC8717635; doi:10.1021/acsnano.1c07381)
Supplement: Supplementary file 1 — nn1c07381_si_001.pdf [file nn1c07381_si_001.pdf]

Supplementary Material for "Nanoconfined Fluids: Uniqueness of Water Compared to Other Liquids"

Fabio Leoni,[*,†] Carles Calero,[‡] and Giancarlo Franzese[‡]

†*Department of Physics, Sapienza University of Rome, P.le Aldo Moro 5, 00185 Rome, Italy*

‡*Secció de Física Estadística i Interdisciplinària–Departament de Física de la Matèria Condensada, Universitat de Barcelona, & Institut de Nanociència i Nanotecnologia (IN2UB), Universitat de Barcelona, C. Martí i Franquès 1, 08028 Barcelona, Spain*

E-mail: fabio.leoni@uniroma1.it.

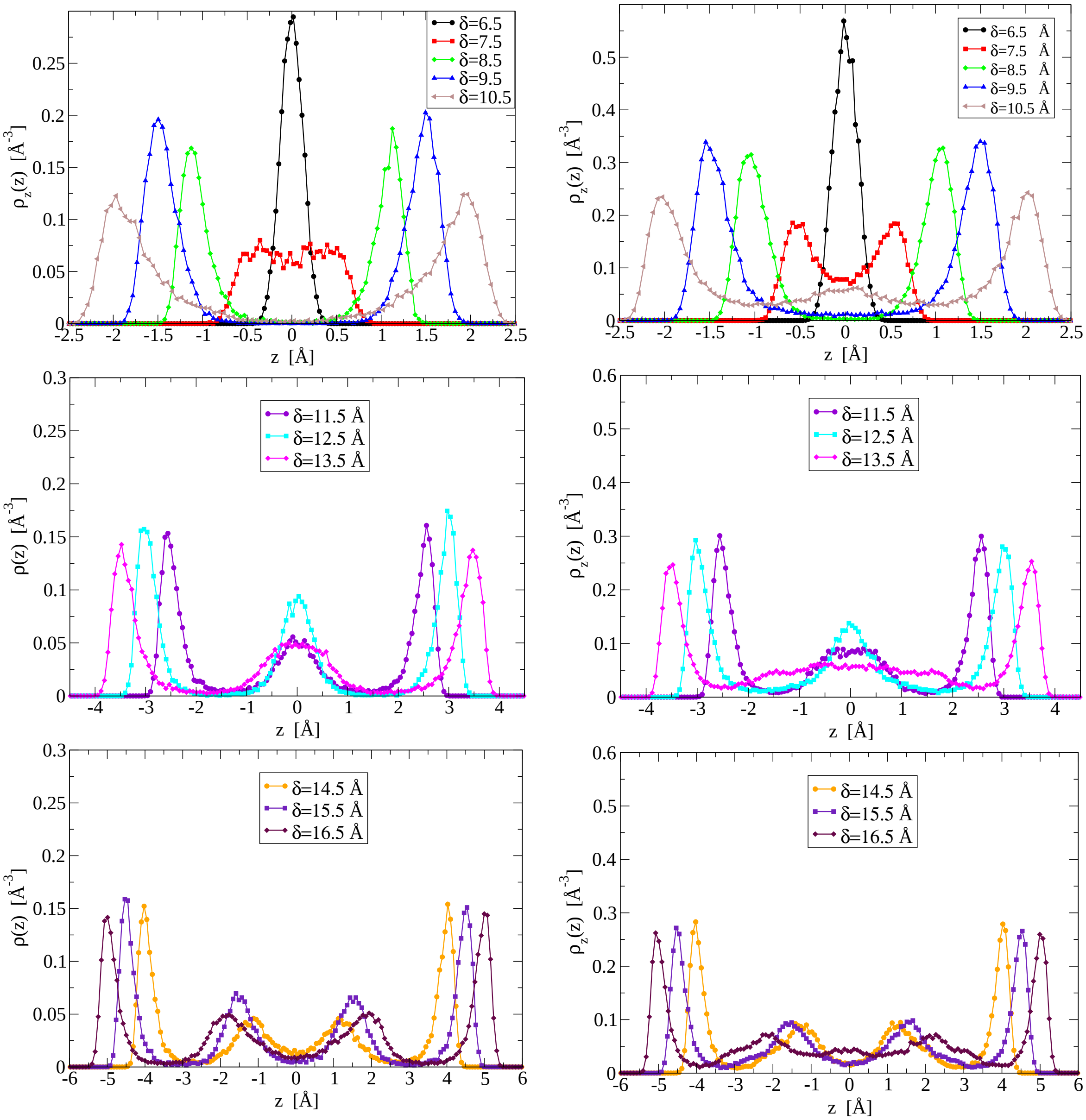

Figure S1: Density profiles $\rho_z(z)$ for isotropic liquids (LJ fluid, left panels; CSW fluid, right panels) in a slit-pore with week fluid-walls interaction, $\epsilon_{w_1} = 0.1$ kcal/mol, for different values of wall-to-wall separation δ. The density ρ_z is calculated along the direction z orthogonal to the walls, within the sub-volume V^s, at $T = 100$ K. The coordinate $z = 0$ marks the center of the pore. For the LJ fluid we set $P_{\text{bulk}} = 1$ atm, corresponding to bulk number-density $\rho_{\text{bulk}} = 0.023$ Å^{-3}. For the CSW fluid we set $\rho_{\text{bulk}} = 0.036$ Å^{-3}.

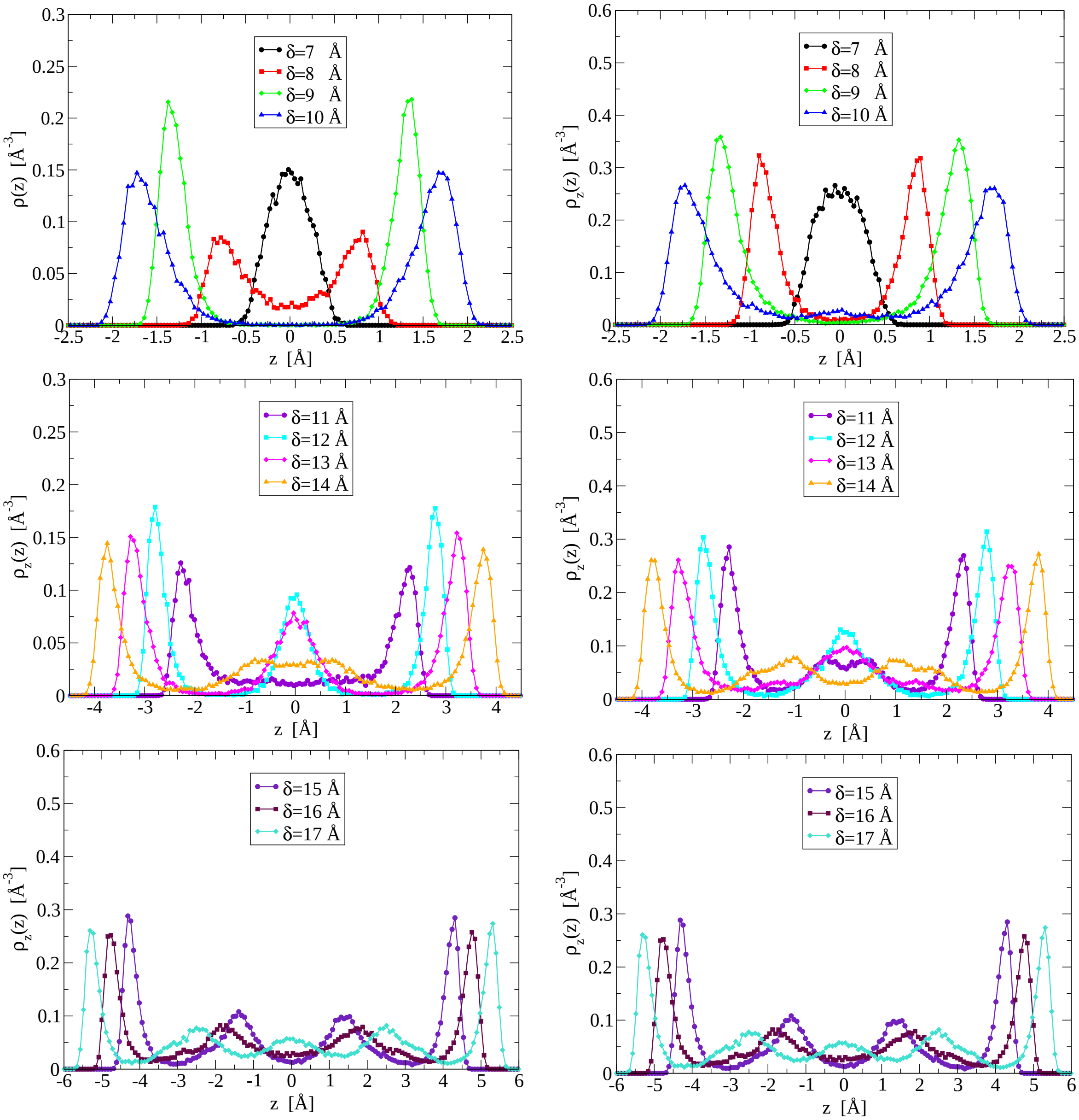

Figure S2: As in Fig. S1, for additional wall-to-wall separation δ for the LJ fluid (left panels) and the CSW fluid (right panels).

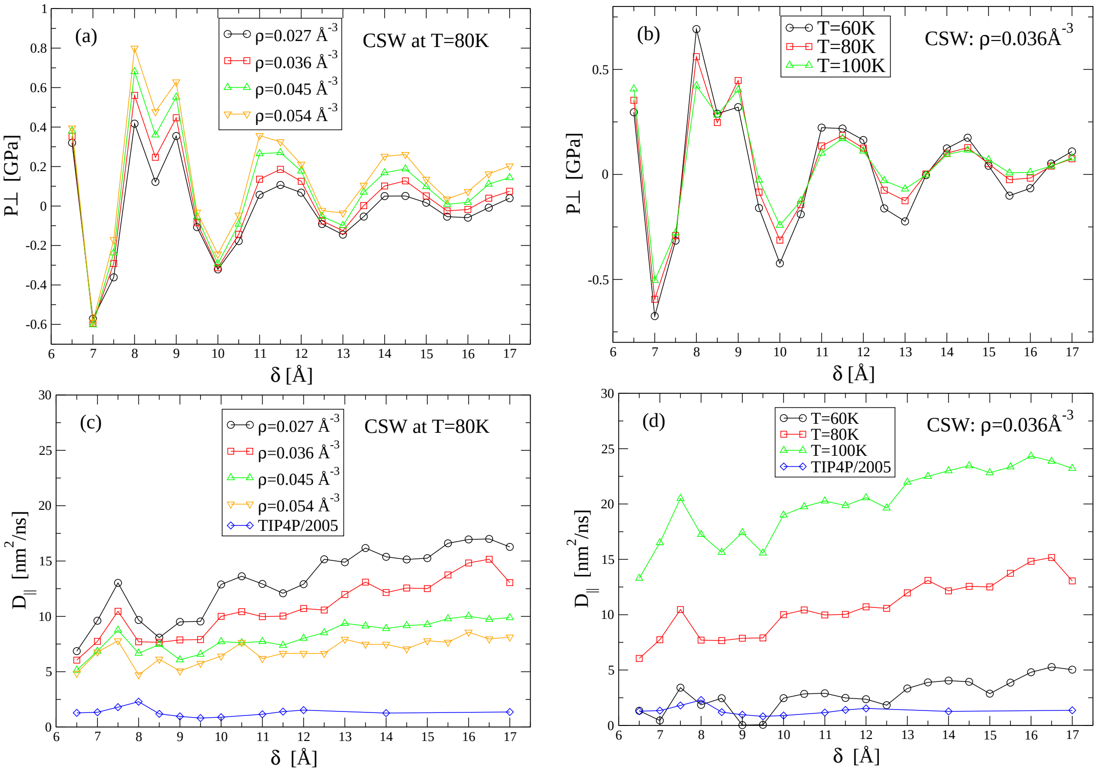

Figure S3: Normal pressure $P_\perp$ and longitudinal diffusion constant $D_\parallel$ inside the confined sub-region as a function of the plate separation δ for the CSW fluid for different densities at fixed temperature (a), (c), and for different temperatures at fixed density (b), (d), respectively. Data for TIP4P/2005-water are at $T = 300$ K and $\rho = 0.033$ Å^{-3}.

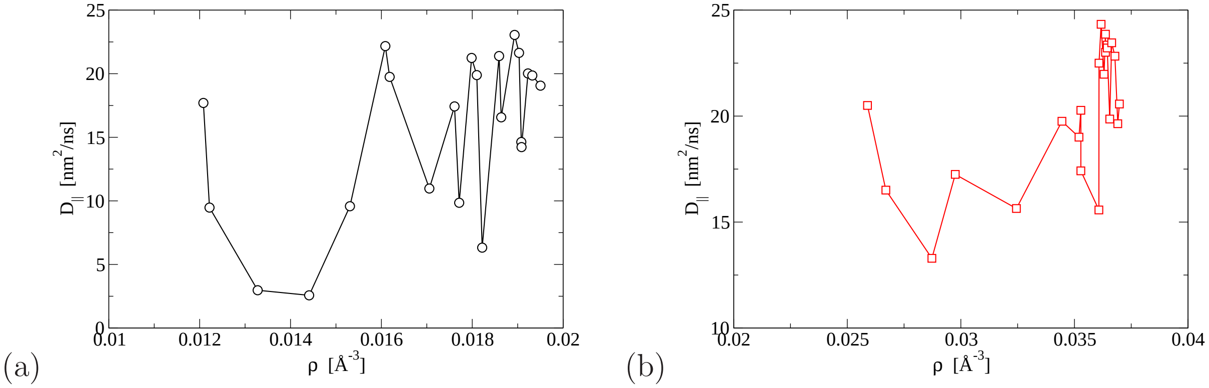

Figure S4: Longitudinal diffusion coefficient $D_\parallel$ as a parametric function of the mean density ρ within the pore, for (a) the LJ (black circles), and (b) the CSW (red squares).

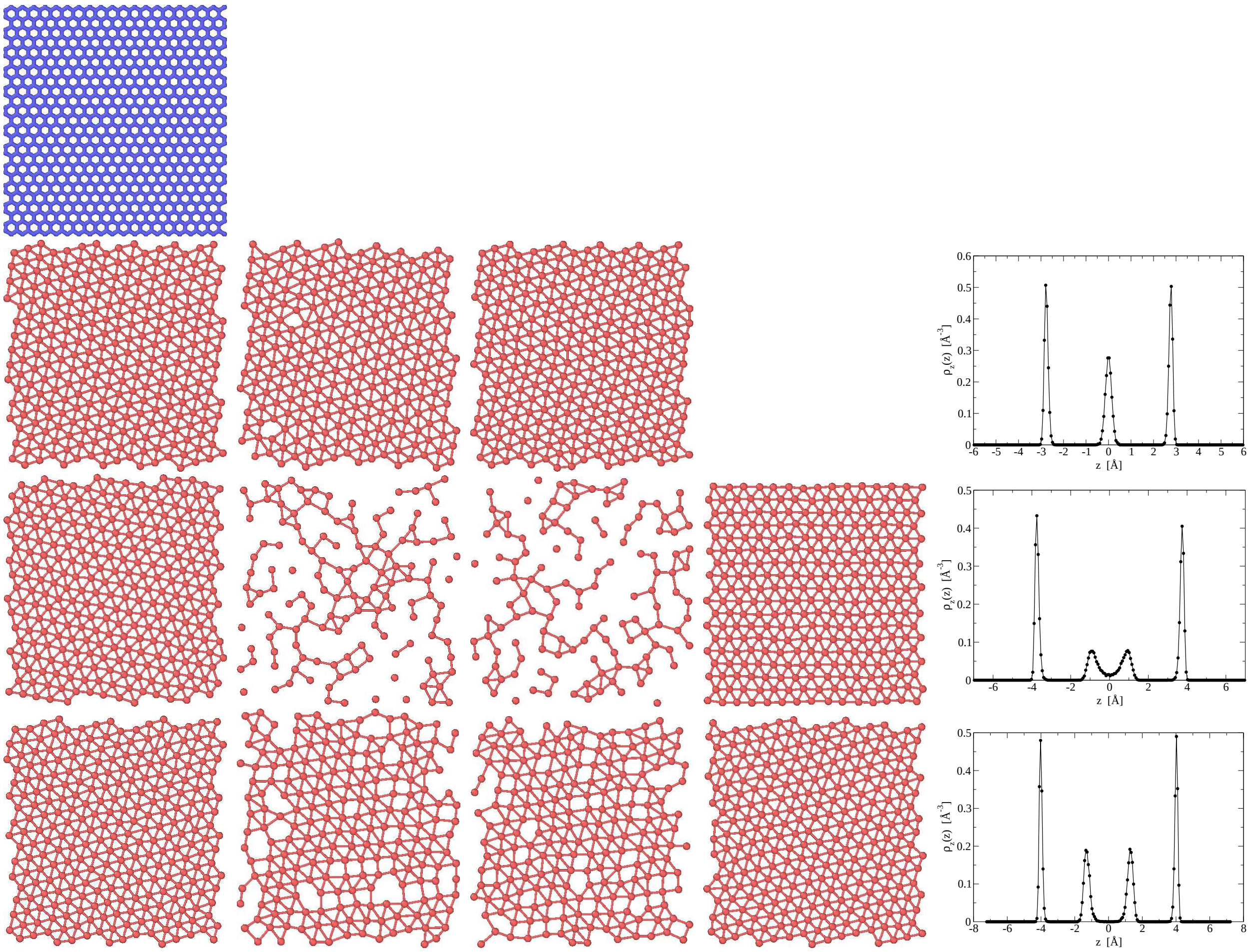

Figure S5: Snapshots of the last frame of the simulated dynamics and density profile (rightmost panels) for the LJ with strong fluid-wall interaction ($\epsilon_{w_2} = 0.48$ kcal/mol). From top to bottom, we show the snapshots of the graphene wall, and the layers formed by the liquid in the confined region for $\delta = 12$ Å, $\delta = 14$ Å, and $\delta = 14.5$ Å. From left to right, the snapshots are associated to layers going from the bottom (for negative z) to the top (for positive z) of the confined region. The ordering in the layers is correlated to the density profile and the parallel diffusivity, as discussed in the main text. Particles closer than 4.5 Å are connected with bonds. This choice allows to link a particle with all the others found within the first minimum of the radial distribution function of the bulk liquid. We observe that the graphene walls exert a molding effect on the LJ liquid, as discussed in.[1]

References

(1) Leoni, F.; Franzese, G. Structural Behavior and Dynamics of an Anomalous Fluid between Attractive and Repulsive Walls: Templating, Molding, and Superdiffusion. *J. Chem. Phys.* **2014**, *141*, 174501.
